# Technology intervention to support caregiving for Alzheimer’s disease (I-CARE): study protocol for a randomized controlled pilot trial

**DOI:** 10.1186/s40814-020-00755-2

**Published:** 2021-01-11

**Authors:** Tyler Braly, Doris Muriathiri, Janetta C. Brown, Britain M. Taylor, Malaz A. Boustani, Richard J. Holden

**Affiliations:** 1 Center of Health Innovation and Implementation Science, Indiana Clinical and Translational Sciences Institute, 410 W. 10th St, Indianapolis, IN 46202 USA; 2 Indiana University Center of Aging Research, Regenstrief Institute, 1101 W. 10th St, Indianapolis, IN 46202 USA; 3 Indiana University School of Medicine, 340 W 10th St #6200, Indianapolis, IN 46202 USA; 4 The Luddy School of Informatics, Computing, and Engineering, Indiana University, 919 E 10th St, Bloomington, IN 47408 USA

**Keywords:** Alzheimer’s disease, Dementia, Brain diseases, Central nervous system diseases, Nervous system diseases, Tauopathies, Neurodegenerative diseases, Neurocognitive disorders, Mental disorders

## Abstract

**Background:**

Informal caregivers of patients with Alzheimer’s disease and related dementias (ADRD) manage a complex spectrum of patient behavioral and psychological symptoms of dementia (BPSD). Mobile health information technologies have quickly become sources for modern social support and chronic disease management. These technologies can improve our understanding of how to care for patients with ADRD and their informal caregivers. A mobile telehealth intervention could help reduce caregiver burden and BPSD.

**Methods:**

This is a pilot randomized controlled trial of 60 dyads of patients living with ADRD and their caregivers, to test the feasibility and estimate the potential effect of the Brain CareNotes (BCN) mobile telehealth system. Participants will be recruited from two health systems. Participants will be randomly assigned to either the BCN intervention arm or usual care comparator. Data will be collected at baseline, 3- and 6-month follow-up. The primary objectives of this trial are to assess feasibility outcomes: (a) recruitment rate, (b) data completion, (c) BCN usability, (d) BCN acceptance, and (e) BCN use and assessed either on an ongoing basis or at 3- and 6-month post-intervention. A secondary objective was to estimate the intervention’s effects on caregiver burden and patient BPSD outcomes at 3 and 6 months, assessed by the Neuropsychiatric Inventory.

**Discussion:**

The study will assess the intervention feasibility and potential effect size of the BCN telehealth system as a potentially scalable and lower-cost solution for addressing the ADRD public health crisis.

**Trial registration:**

Clinical Trials. NCT03119259. Registered on April 18, 2017.

## Background

Caring for someone with Alzheimer’s disease and related dementias (ADRD) is associated with higher rates of psychological morbidity and burden, social isolation, financial hardship, and deterioration of physical health [1]. Much care is administered by informal caregivers, who are family or friends who provide regular care outside of a formal professional role to those people who unable to function independently [2]. In 2019, 16.3 million informal caregivers provided 18.6 billion hours of unpaid care to the 5.8 million Americans living with ADRD [3]. This care has been valued at a nationwide contribution of $244 billion [3]. Informal caregivers of patients with ADRD manage a complex spectrum of patients’ behavioral and psychological symptoms related to dementia (BPSD); BPSD are major contributing factors to caregivers’ burden and adverse health outcomes [2, 4]. Excessive caregiver burden leads to higher unplanned hospitalization rates and poorer quality of life [5, 6]. The National Alzheimer’s Project Act recognizes the need for interventions that “enable family caregivers to continue to provide care while maintaining their own health and well-being.” [7] A variety of interventions have been shown to be effective in reducing both BPSD symptoms and caregiver burden [8–10]. However, with the threefold increase of the number of Americans living with ADRD projected for 2050, an effective intervention would also need to be easily scalable if it were to meaningfully address these problems [11].

Mobile health and telehealth technologies offer a means of addressing these issues [12]. Internet-based technologies have been shown to be an effective means of intervention for older adults in general [13]. Although a recent systematic review suggests potential benefits of mobile applications for persons with cognitive impairment, telehealth applications, which connect patients and informal caregivers to healthcare professionals, were rarely studied in these populations, especially among persons with ADRD and their informal caregivers [14]. However, factors such as age, education, and cognitive impairment can make utilizing this approach less effective, thus highlighting the need for effective design and careful testing for usability, acceptability, and actual use [15–17].

## Methods

### Study design

**Fig. 1:**
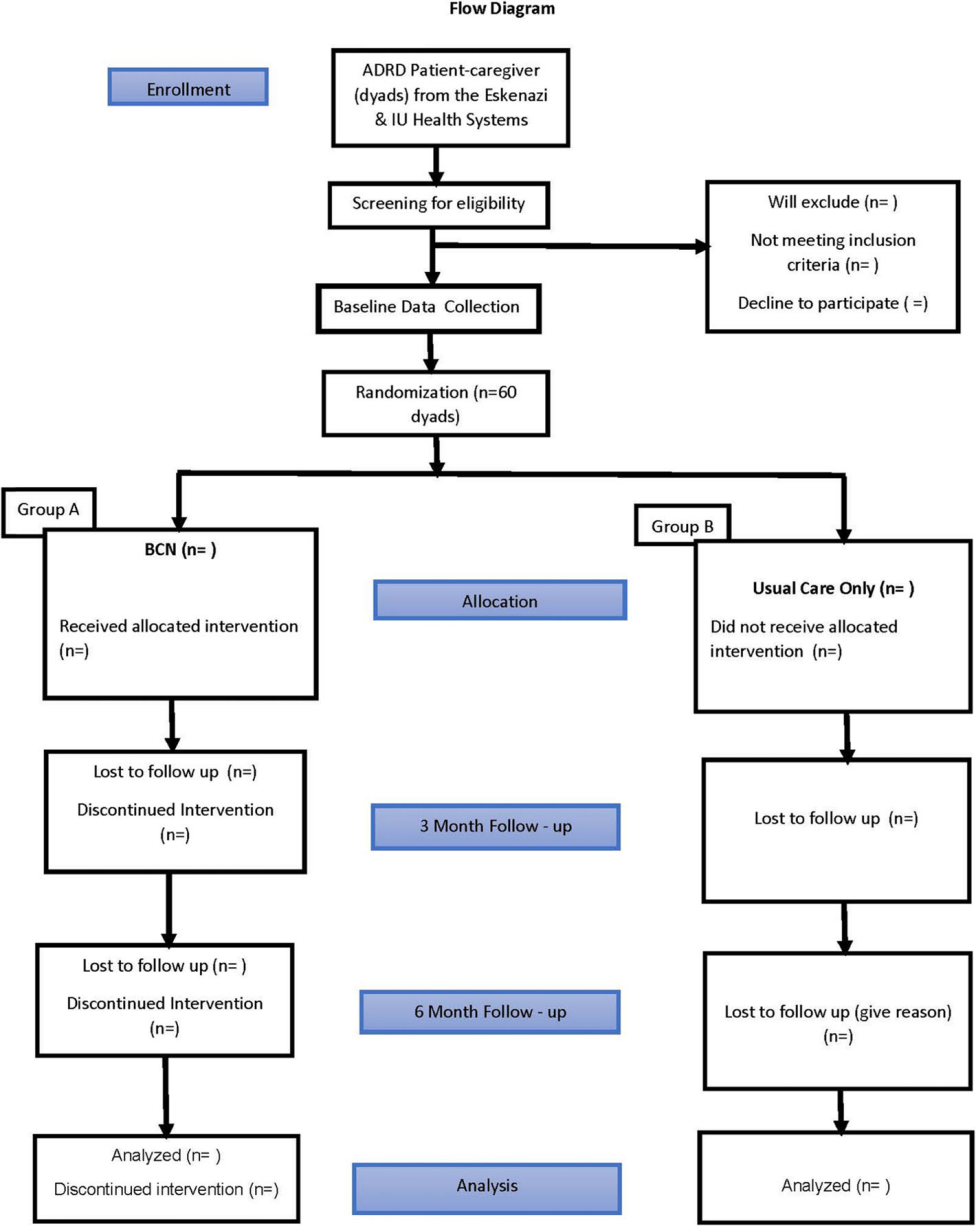
Study flow diagram

This is a randomized controlled pilot trial of 60 ADRD patient-caregiver dyads to test the feasibility and estimate the potential effect on caregiver burden and BPSD of the mobile telehealth technology Brain CareNotes (BCN) (see Fig. 1). Participants will be recruited from two health systems and randomly assigned to either the BCN intervention arm or usual care comparator. Study participation will last for 6 months.

The primary goal of this trial is to test the feasibility outcomes of: (a) recruitment rate, (b) data completion, (c) BCN usability, (d) BCN acceptance, and (e) BCN use, assessed either on an ongoing basis or at 3- and 6-month post-intervention. Secondarily, the trial will estimate the intervention’s effect on outcomes of caregiver burden and patient BPSD at 3 and 6 months, assessed by the Neuropsychiatric Inventory.

The target sample was chosen to mirror a dyad-per-research-staff enrollment rate over the course of the trial comparable to what would be needed for a larger trial powered to detect intervention effects on caregiver and patient outcomes. This sample size of 60 dyads was judged to produce adequately precise confidence intervals around estimates of the feasibility outcomes [18]. With this sample size, there is also 80% power to detect a large effect size on caregiver and patient outcomes of 0.75 standard deviations between the two groups.

### Setting and participants

This study has a planned enrollment of 60 patient-caregiver dyads. There will be a recruitment of 30 dyads in the intervention arm and 30 dyads in the usual care comparator. Dyads will be recruited from two health systems in Indiana, USA: (1) geriatric clinics specializing in patients with cognitive impairment at Eskenazi Health, a safety net public healthcare system serving Indianapolis, Indiana; and (2) primary care clinics at Indiana University (IU) Health, a nonprofit academic healthcare system. All dyads contain both a patient with a diagnosis of ADRD and an informal caregiver who manages their care. Patients with ADRD and their caregivers are key intervention targets because they are at high risk of BPSD- and BPSD-related adverse effects on caregivers, respectively. BPSD in this population are a top contributor to hospitalization, nursing home placement, and new disease or disability.

### Description of intervention

The primary intervention of this study is the delivery of the BCN mobile telehealth application (app), a mobile app for informal caregivers of patients with ADRD. BCN delivers 24/7 psychoeducation and caregiver support. In addition, there are caregiver-reported assessment of patient BPSD and tools for self-management and caregiver-clinician communication. BCN provides 24/7 access to information on caregiving delivered through “bite-sized” CareNotes, which convert evidence-based best practices into simple, single sentences offering practical advice. For example, advice for everyday care during mealtime is to “Offer regular drinks to avoid dehydration.” Personal, relatable stories are used to anchor information modules and show how caregiving challenges are universal across cultures. These stories were prepared by dementia care professionals based on past experiences with actual patients. BCN provides patient and caregiver self-assessment tools in the form of the Healthy Aging Brain Care (HABC) Monitor, a clinically validated test of caregiver perceptions of cognitive, functional, and behavioral symptoms and caregiver distress [19]. This assessment is delivered through the mobile app and securely communicates the results to practicing clinicians. The results of completed assessments are available in real time to clinicians, who presently receive daily alerts indicating new completed assessments and are expected to review and document all new assessments. The results of these assessments are used to personalize the CareNote advice cards given to the caregiver. In this regard, the recommended CareNote advice cards presented to a user are algorithmically selected based on individual item scores on the assessment. Caregivers are also given the ability to browse, save, edit, and arrange recommended advice cards. Lastly, the app offers a secure messaging system that connects caregivers and clinicians. The messaging is available 24/7 to both parties, although clinicians are expected to use the messaging during work hours. In the present delivery of the intervention, community health workers with training in coaching caregivers of persons with dementia on behavioral symptom management receive a daily morning email informing them whether new messages have been received, with the expectation they will check for and respond to messages throughout the day. Regardless of who initiates the messaging, the responsible clinician communicates with caregiver users through the BCN secure messaging system. When a user completes a BPSD self-assessment via the BCN app, this is treated as an assessment comparable to phone or in-person assessment by a clinician and can therefore be used in place of those. However, the use of BCN does not replace other services or visits for purposes other than BPSD assessment.

Caregivers in dyads randomized to BCN will receive a HIPAA compliant BCN application link to download the mobile app to their personal mobile device if the device meets minimum technical requirements. If the caregiver does not have a mobile device meeting minimum requirements, a smartphone device with unlimited network data plan from AT&T and the BCN app installed will be provided by research staff for the duration of the study.

A research assistant (RA) will orient participants to the device, provide training on the BCN software, and troubleshoot technical issues in person or remotely. Participants will receive technical support by phone or electronically by members of the study team, during work hours. They will also be provided paper and in-app help manuals. Hardware, software, and connectivity check-ups will be provided by study research personnel as needed.

### Description of usual care comparator

All patients and informal caregivers in both the intervention and usual care arms will receive usual primary care provided by Eskenazi Health or IU Health. Additionally, usual care for patients with ADRD at Eskenazi Health includes care in the Aging Brain Care (ABC) clinical program, delivered by an interdisciplinary team including expertise in geriatric medicine, social work, and nursing, led by a care coordinator according to an individualized care plan.

### Inclusion/exclusion criteria

Participant eligibility criteria are the patient has received a diagnosis of possible or probable Alzheimer’s disease or related dementia from a physician at Eskenazi Health or IU Health, the caregiver is at least 18 years of age and does not have a visual impairment significant enough to interfere with their ability to use BCN, both patient and caregiver are community-dwelling in central Indiana, and both patient and caregiver are willing to participate according to the study protocol. Exclusion criteria are the patient or their informal caregiver does not have the ability to communicate in English.

### Recruitment and retention

Participants will be recruited from two large healthcare delivery systems in Indiana. Dyads will be screened using the inclusion/exclusion criteria listed above by a RC or RA, using each system’s respective electronic health records system. Eligible dyads will be called by RAs and RCs to see if they are interested in study participation. To minimize the burden of study participation, subjects will receive the option of remote data collection by phone and data collection at a location convenient to the participants, the default for the latter being the patient’s home. Research staff will be in regular contact with participants to maintain engagement.

Due to variation between usual care in the two healthcare systems, study site will be recorded as an indicator to include as a covariate in analyses.

A challenge to the success of the proposed study is achieving adequate enrollment and retention. Enrolling older adults including persons with ADRD is uniquely challenging and retention is complicated by an approximate 8% annual attrition due to death alone. This study will also require enrolling two individuals per dyad, who must consent to a 6-month study period and three assessments.

### Ethics and informed consent

This trial was approved by the Indiana University Institutional Review Board (IRB# 1606267154) and is registered on ClinicalTrials.gov (NCT03119259). Both health systems approved the studies through their internal approval system. The two health systems do not have independent IRBs and instead have an arrangement with the university IRB for their studies. Informed consent will be obtained from the patient or legally authorized representative and separately from the caregiver by a trained RA or RC. Individuals will receive verbal and written information about consent and HIPAA authorization, including answers to all their questions and a copy of the documents. Each individual or their surrogate must provide verbal or written documented consent and HIPAA authorization to participate. Consent by proxy will be used for a patient if any of the following three conditions is met:

1. The patient has an MMSE score = 17
2. The patient is unable to answer any of the following 4 questions correctly:
  - What do you understand is wrong with your brain health right now?
  - Can you please explain in your own words the choices I have presented to you?
  - If you decide to participate in this study, what good things might happen and what harm might occur?
  - Can you please explain how you decided to participate (or not participate) in this study?
3. The patient’s provider states that the patient does not have the capacity to consent to participation in the study based upon the provider’s knowledge of the patient.

When consent by proxy is used, research personnel will also seek the assent of the patient.

Risks of trial participation are expected to be minimal and rare. Participants or their surrogates can choose to withdraw from the study at any time, for any reason. All research personnel are IRB-approved and appropriately trained on confidentiality and enrollment procedures. Eligible and enrolled participants’ data will be collected, shared, and maintained in accordance with the IU IRB and HIPAA guidelines. Only IRB-approved study team members will have access to the collected data. In addition, all consented participants will be assigned a unique study identifier upon enrollment. The data will be stored in a secure Research Electronic Data Capture (REDCap) database on a password-protected IU server. REDCap was specifically developed around HIPAA security guidelines.

### Randomization

This randomized controlled pilot trial utilizes a randomization block to sort subjects into the BCN intervention or usual care comparator group in a 1:1 manner. The randomization blocks were created by the PI, approved by an independent biostatistics faculty member, and loaded in REDCap by the team biostatistician. Masking/blinding to randomization was not implemented.

### Assessments and outcomes

Assessments will be performed at baseline pre-randomization and at 3- and 6-month follow-ups post-randomization, as shown in Table 1. The caregiver is contacted by phone to schedule each appointment. The feasibility outcomes assessed are (a) recruitment rate, (b) data completion, (c) BCN usability, (d) BCN acceptance, and (e) BCN use. The outcomes measured to estimate potential effect sizes of the intervention are caregiver burden and patient BPSD. Assessments will be performed by appointment on the phone or in person. Participants will receive $25 per assessment and up to $75 in total for the entire project.

**Table 1:** Assessment schedule

	Patient/caregiver demographics	Neuropsychiatric inventory (NPI)	Single-item literacy screener	System usability survey	Behavioral intervention – BCN acceptance
Baseline	✓	✓	✓		
Month 1				✓	✓
Month 3		✓		✓	✓
Month 6		✓		✓	✓

Recruitment rate and data completion will be collected via timestamped research records in REDCap on an ongoing basis throughout the duration of the trial. Recruitment rate will be collected continually via timestamped research records in REDCap and computed at the end of the accrual period by calculating # approached, # agreeing, # consenting, and # consenting/# approached. We will also calculate the monthly rate. Data completion will be collected continually in the REDCap study database and computed after the final assessment by calculating # of complete assessments per consenting participant/total possible assessments. We will separately compute missing data and attrition for all causes vs. death in the intervention vs. control groups. A data manager will assist in feasibility data capture and reporting.

BCN usability will be assessed in the BCN intervention group at 3 and 6 months by the Simplified System Usability Scale (SUS) [20], a 10-item questionnaire administered to caregivers. The Simplified SUS contains statements about usability (e.g., “Learning to use Brain CareNotes was quick for me”) answered on a 5-point response scale (strongly disagree to strongly agree) and is modified from the original validated SUS [21]. BCN acceptance will be assessed in the BCN intervention group at 3 and 6 months as the mean score on the Behavioral Intention Questionnaire administered to caregivers. It is a 4-item questionnaire and uses a 7-point response scale from 0 (not at all) to 6 (a great deal) [22, 23]. Behavioral intention is the accepted measure of technology acceptance. BCN use will be collected continually from captured server activity for log-in, survey initiation, survey completion, use of CareNotes advice cards, and messages sent and received. We will compute daily, weekly, and monthly use rates for each use type and for any use at 3 and 6 months.

The Neuropsychiatric Inventory (NPI) will be administered to caregivers, measuring caregiver-reported patient behavior and obstacles to caregiving [24]. Informal caregiver burden will be assessed by calculating the NPI-Caregiver Distress Score (possible range from 0 to 60) from the NPI assessed at baseline, 3 months, and 6 months. Patient BPSD will be assessed by calculating the NPI total score (possible range from 0 to 144) from the NPI at baseline, 3 months, and 6 months.

### Additional data collection

Demographic data for both the caregiver and patient will be collected after enrollment, including age, gender, race, ethnicity, education level, employment status, annual household income, living situation, and insurance status. The total hours over the previous 2 weeks the caregiver spent with the patient and the duration of time the caregiver has been caring for the patient will be recorded. Medical data for the patient will also be collected. Health literacy will be assessed by the Single-Item Literacy Screener (SILS) [25].

### Analyses

The completeness and distributions of all variables will be checked. We will check the shape of continuous distributions to detect strong deviation from normality and will apply normalizing transformations as needed for highly non-normally distributed variables (e.g., NPI). Internal consistencies of scale scores will be assessed with Cronbach’s coefficient alpha. Two-sample *t* tests and chi-squared tests will be used to compare demographic and clinical characteristics of the participants between the two groups at baseline.

The primary aim is to evaluate the feasibility of BCN as an intervention. The following variables will be determined to test this:

- A recruitment rate per month
- Completion on primary outcome measures
- Mean score on the 10-item SUS [21]
- BCN acceptance on the Behavioral Intention 3-item scale [17]
- % weekly BCN use

Analyses for (a) and (b) will be descriptive in nature, and no statistical tests will calculated. For (c), we will calculate the mean and standard deviation for the composite SUS to calculate the 90% confidence interval. For (d) and (e), we will calculate the proportion of patients with BCN acceptance and weekly BCN use and then calculate 90% confidence intervals.

A secondary aim is to estimate the effect of the BCN intervention on caregiver burden. A linear mixed model will be used to compare the two groups on the mean total NPI-Caregiver Distress Scores at 3 and 6 months, using an analysis-of-covariance (ANCOVA) style of model in which the baseline measure of the outcome variable is included as a fixed effect. Time (3 and 6 months), intervention (BCN vs. usual care), and the interaction between time and intervention will be included in the model to test for group differences at each time point. If NPI is highly positively skewed, as we have observed in prior research, we will perform a transformation on NPI, such as log (NPI+1), to satisfy model assumptions. All analyses will be intention to treat (ITT), and additional sensitivity analyses will assess the effect of imputing missing outcomes using several multiple imputation techniques (both missing at random and not missing at random).

Another secondary aim is to estimate the effect of the BCN intervention on BPSD. Analyses will be performed as described above for caregiver burden, except the outcome will be the NPI total score.

### Data and safety monitoring plan and board

The Principal Investigators and an independent Safety Officer will be responsible for data and safety monitoring, with support from the team’s data manager and biostatistician. Research staff will prepare for them a formal report communicating the study’s enrollment progress, data collection, and participant safety. Investigators will meet at least every 2 weeks with the study team to review ongoing study progress and participant safety and will review and discuss every reportable event within 48 h. The frequency for reviewing data in this study differs according to the type of data and is summarized in Table 2.

**Table 2:** Frequency of review

Data type	Frequency of review		
	Each occurrence	Quarterly	Annually
Self-reported adverse events	X		
Serious adverse event meeting prompt reporting criteria	X		
Summary of serious adverse events		X	
Protocol violations/noncompliance		X	
Summary of adverse events			X
Subject accrual/randomization		X	
Withdrawal rates		X	
Subject complaints		X	
Compliance to interventions		X	
Out of range data			X
Reassessment of risk-to-benefit			X

All data for each participant will be compiled in a single REDCap database. REDCap offers secure access, regular backup, data validation, and structured data collection via mobile devices for all interviewer-administered assessments. Medical and care process data will be collected from electronic medical records and entered into the REDCap database. Data on device use will be obtained via server queries and uploaded to REDCap. Recruitment process data will be directly input into REDCap by research staff. A staff biostatistician or data manager will perform the merging of all data streams using unique identifiers assigned to the study participants. They will regularly review data for data entry errors and other irregularities.

### Special considerations

A challenge for this study is ensuring that those randomized to receive BCN have adequate ongoing access to BCN technology. We have addressed this challenge by applying iterative user-centered design and testing of BCN, paying consideration to the needs and abilities of middle-aged and older adult caregivers. The user-centered design and testing process included a study of processes and unmet needs of the current clinical program [26], followed by the app designers convening individual and group meeting of caregivers and clinicians to discuss needs and design ideas. Thereafter, we collected initial feedback from caregivers on screen mockups and features, followed by a brief field test of the application with 11 caregivers who used BCN on an Android device. In the post-test usability interview, we assessed caregivers’ perceived usefulness, usability, desirability, and credibility of the app and adjusted the app to address problems. When possible, the BCN app is installed directly on the caregiver’s smartphone. Those dyads that need a smartphone are provided a smartphone with an unlimited data plan for the duration of the study. We will document the proportion that requires such additional assistance, as this may ultimately affect the real-world implementation of an intervention like BCN. Assistance with software installation and subsequent technical support needs are provided for all study participants. We have also budgeted for network and development support to monitor and remediate (e.g., with patches) any emergent technical malfunctions, operating system changes, or security vulnerabilities during the study. These steps are important to reducing the risks of systematically excluding more vulnerable participants and creating or increasing inequalities in technology studies [27].

## Discussion

Reducing caregiver burden and minimizing BPSD are important for improving wellbeing and reducing other health risks of ADRD caregiving. Mobile and telehealth technology applications are promising interventions to improve these outcomes in a scalable manner [14]. However, before a technology is deployed to a large sample or in clinical practice, it must be evaluated for usability, acceptance, and potential efficacy. This is especially needed to avoid the potential for harm or waste of effort when the technology is intended for older and cognitively impaired individuals, those who provide them informal care, or other vulnerable groups. The present randomized controlled pilot study aims to collect the necessary evidence to determine the viability of the BCN telehealth system.

If found to be feasible and potentially efficacious, BCN and other mobile technologies have the potential to be scalable. An app such as BCN could be shared with a broad pool of study participants or members of the public through existing public app marketplaces and can be downloaded on personal devices or accessed via a website. Although the cost of an intervention such as BCN would have to be further assessed, once it is built, major anticipated technology costs include software and security maintenance. Additional costs may be related to time spent by a community health worker reviewing data entered by caregivers into the app and participating in secure messaging. A telehealth system also allows collaborative, interdisciplinary care from individuals as diverse as geriatricians, care coordinators, nurses, social workers, community health workers, informal caregivers, and community members. The breadth of involvement and the low barriers to access permit contributions to care from larger “workforces,” for example, community health workers, volunteers, or a network of informal caregivers. This is an important advance given anticipated increases in the demand for ADRD care and the limited number and cost of geriatricians and memory care specialty services. Another, more prospective potential for a telehealth system is to deliver care or health coaching through or assisted by artificial intelligence.

Overall, if successful, this pilot study can serve as a test case for a new way of delivering better care to patients with ADRD and their caregivers.

### Trial status

Enrolling. Date recruitment began: 11/2017. Approximate date recruitment will be completed: 04/30/2021. Latest protocol version approved 05/14/2020 (Amendment 011) Indiana University IRB study # 1606267154.

## Data Availability

Not applicable.

## Abbreviations

ABC: Aging brain care
ADRD: Alzheimer’s disease and related dementias
BCN: Brain CareNotes
BPSD: Behavioral and Psychological Symptoms of Dementia
HIPAA: Health Insurance Portability and Accountability Act
IRB: Institutional Review Board
IU: Indiana University
HABC Monitor: Healthy Aging Brain Care Monitor
RA: Research assistant
RC: Research coordinator
REDCap: Research Electronic Data Capture
SUS: System Usability Scale

## References

[1] National Institute on Aging. Next steps for research on informal caregiving. https://www.nia.nih.gov/sites/default/files/2017-08/gerald-summary_11-21-14_0.pdf.

[2] Schulz R, Martire LM (2004). Family caregiving of persons with dementia: prevalence, health effects, and support strategies. Am J Geriatr Psychiatry Off J Am Assoc Geriatr Psychiatry.

[3] Alzheimer’s Association (2020). 2020 Alzheimer’s Disease Facts and Figures. Alzehimers Dement.

[4] Schulz R, Beach SR (1999). Caregiving as a risk factor for mortality: the Caregiver Health Effects Study. JAMA.

[5] Schubert CC (2008). Acute care utilization by dementia caregivers within urban primary care practices. J Gen Intern Med.

[6] Fowler NR (2012). Association between cognitive decline in older adults and use of primary care physician services in Pennsylvania. J Prim Care Community Health.

[7] Alzheimer’s Association National Plan Care and Support Milestone Workgroup (2016). Report on milestones for care and support under the U.S. National Plan to Address Alzheimer’s Disease. Alzheimers Dement.

[8] Callahan CM (2006). Effectiveness of collaborative care for older adults with Alzheimer disease in primary care: a randomized controlled trial. JAMA.

[9] LaMantia MA (2015). The aging brain care medical home: preliminary data. J Am Geriatr Soc.

[10] Parker D, Mills S, Abbey J (2008). Effectiveness of interventions that assist caregivers to support people with dementia living in the community: a systematic review. Int J Evid Based Healthc.

[11] Sloane PD (2002). The public health impact of Alzheimer’s disease, 2000–2050: potential implication of treatment advances. Annu Rev Public Health.

[12] Changizi M, Kaveh MH. Effectiveness of the mHealth technology in improvement of healthy behaviors in an elderly population—a systematic review. mHealth. 2017;3. 10.21037/mhealth.2017.08.06 PMC5803024 29430455

[13] Martínez-Alcalá CI, Pliego-Pastrana P, Rosales-Lagarde A, Lopez-Noguerola J, Molina-Trinidad EM. Information and communication technologies in the care of the elderly: systematic review of applications aimed at patients with dementia and caregivers. JMIR Rehabil Assist Technol. 2016;3. 10.2196/rehab.5226 PMC5454565 28582258

[14] Bateman DR (2017). Categorizing health outcomes and efficacy of mHealth Apps for persons with cognitive impairment: a systematic review. J Med Internet Res.

[15] Holden RJ, Boustani MA. Design and usability methods: gile innovation and evaluation of interventions for patients and families. In: The Patient Factor: Theories and Methods for Patient Ergonomics: CRC Press; 2020.

[16] Marziali E, Garcia LJ (2011). Dementia caregivers’ responses to 2 Internet-based intervention programs. Am J Alzheimers Dis Other Dement.

[17] Finkelstein J, et al. Enabling patient-centered care through health information technology. Evid ReportTechnology Assess. 2012:1–1531. PMC4781073 24422882

[18] Hertzog MA (2008). Considerations in determining sample size for pilot studies. Res Nurs Health.

[19] Monahan PO (2012). Practical clinical tool to monitor dementia symptoms: the HABC-Monitor. Clin Interv Aging.

[20] Holden RJ (2020). A simplified system usability scale (SUS) for cognitively impaired and older adults. Proceedings of the International Symposium on Human Factors and Ergonomics in Health Care.

[21] Bangor A, Kortum PT, Miller JT (2008). An empirical evaluation of the System Usability Scale. Int J Human–Computer Interact.

[22] Holden RJ, Brown RL, Scanlon MC, Karsh B-T (2012). Pharmacy workers’ perceptions and acceptance of bar coded medication technology in a pediatric hospital. Res Soc Adm Pharm RSAP.

[23] Holden RJ, Brown RL, Scanlon MC, Karsh B-T (2012). Modeling nurses’ acceptance of bar coded medication administration technology at a pediatric hospital. J Am Med Inform Assoc JAMIA.

[24] Cummings JL (1994). The Neuropsychiatric Inventory: comprehensive assessment of psychopathology in dementia. Neurology.

[25] Morris NS, MacLean CD, Chew LD, Littenberg B (2006). The Single Item Literacy Screener: evaluation of a brief instrument to identify limited reading ability. BMC Fam Pract.

[26] Heiden SM, Holden RJ, Alder CA, Bodke K, Boustani M (2017). Human factors in mental healthcare: a work system analysis of a community-based program for older adults with depression and dementia. Appl Ergon.

[27] Holden RJ, Toscos T, Daley CN. Researcher reflections on human factors and health equity. In: Advancing Diversity, Inclusion, and Social Justice through Human Systems Engineering: CRC Press. p. 51–62.

